# Discriminating established cardiovascular disease using a novel multiterritory ultrasound plaque burden measure (wTPT): findings from the P-SONAR study

**DOI:** 10.1186/s12872-026-05807-8

**Published:** 2026-04-09

**Authors:** Gunnar Austad, Jonn Terje Geitung, Owen Thomas, Serena Tonstad

**Affiliations:** 1 Department of Preventive Cardiology, Oslo University Hospital, Oslo, N-0424 Norway; 2 Faculty of Medicine, University of Oslo, Oslo, Norway; 3 Department of Radiology and Nuclear Medicine, Akershus University Hospital, Lørenskog, N-1478 Norway; 4 Health Services Research Unit, Akershus University Hospital, Lørenskog, N-1478 Norway

**Keywords:** Subclinical atherosclerosis, Cardiovascular disease, Plaque burden, Cardiovascular risk, Ultrasonography, Peripheral artery disease, NORRISK-2, Risk prediction

## Abstract

**Objective:**

Ultrasound-imaging of subclinical atherosclerosis may refine cardiovascular (CV) risk assessment, but quantification methods vary and often include carotid arteries only. Because atherosclerosis is multiterritory, global (carotid–femoral) plaque quantification may better reflect systemic burden. We examined whether a novel multiterritory measure, weighted total plaque thickness (wTPT), better discriminates established CV disease than traditional risk factors and plaque measures.

**Methods:**

In 5 410 participants (59.8 ± 8.1 years; 53.0% women) from the Prospective Screening Of Non-invasive Atherosclerosis Risk study, plaque burden was assessed across 12 carotid and femoral segments using wTPT, maximal plaque thickness (MPT), plaque count, and number of arteries with plaque. Discrimination of prior CV disease was evaluated using c-statistics, net reclassification improvement (NRI), and integrated discrimination improvement (IDI).

**Results:**

Global wTPT showed the strongest discrimination of established CV disease (c-statistic 0.85 [95% CI: 0.83–0.87]), outperforming alternative plaque measures (*p* < 0.001). Adding global wTPT to risk factors improved discrimination from 0.84 to 0.89 (95% CI: 0.87–0.91), NRI 0.71 (95% CI: 0.57–0.84) and IDI 0.058 (95% CI: 0.004–0.071). Femoral wTPT outperformed carotid wTPT (*p* < 0.001), and global wTPT exceeded both (*p* = 0.014). Across wTPT quartiles, odds of prior CV disease increased stepwise: 2.0 (95% CI: 0.89–4.4), 4.2 (95% CI: 2.0–8.6), and 10.0 (95% CI: 5.0–20.1) versus the lowest quartile.

**Conclusion:**

Global wTPT showed superior discrimination of established CV disease and added substantial incremental discriminative value beyond standard risk factors. These findings support wTPT as a marker of systemic atherosclerosis and a strong candidate for future outcome-based risk refinement. If prospectively validated, wTPT may contribute to improved risk stratification by identifying individuals with a high systemic atherosclerotic burden.

**Trial registration:**

Clinicaltrials.gov. Identifier: NCT06933745. Registered 22 april 2025.

**Graphical Abstract:**

**Figure:**
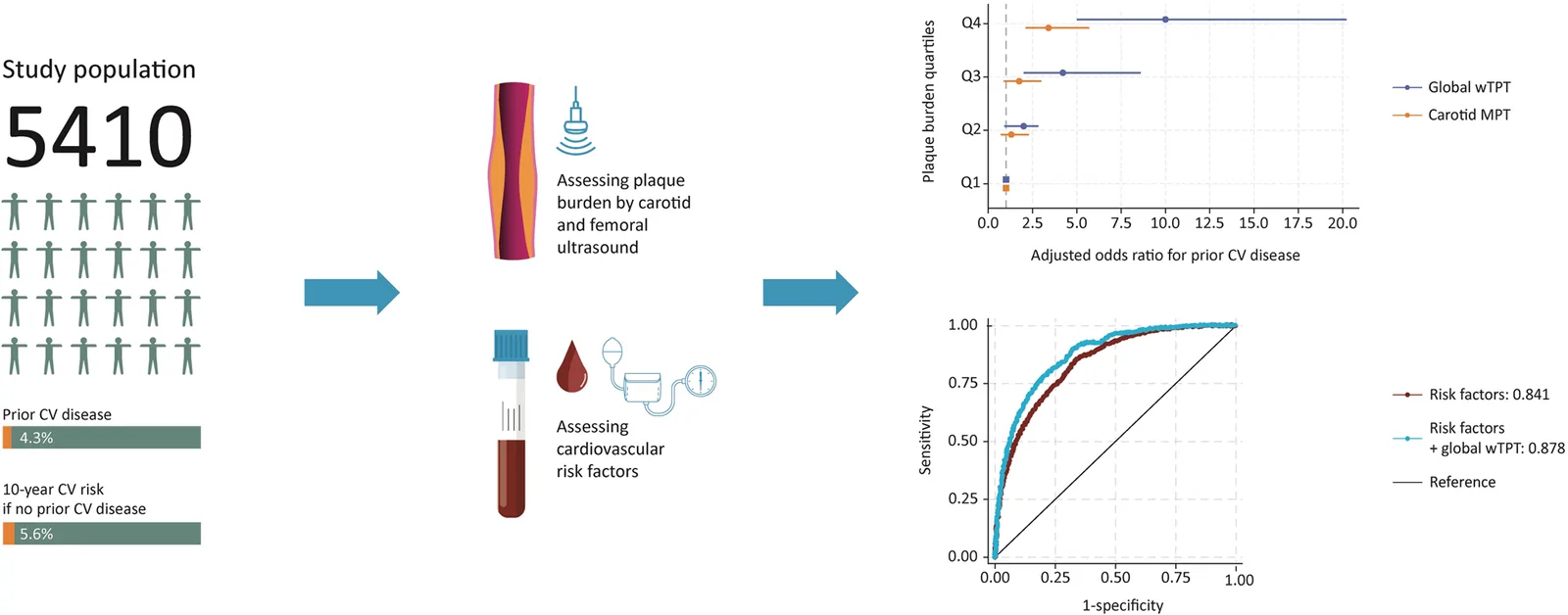

**Supplementary Information:**

The online version contains supplementary material available at https://doi.org/10.1186/s12872-026-05807-8.

## Introduction

The majority of deaths due to cardiovascular (CV) disease are preventable by addressing key risk factors [1]. Early identification of individuals at highest risk is essential for effective prevention. Although risk calculators based on demographic and clinical variables form the foundation of CV disease prevention, they are increasingly recognised as imprecise [2], in part due to the dominant influence of age in the development of atherosclerosis [3]. Furthermore, the prevalence of subclinical atherosclerosis is high even among individuals classified as low risk [4]. 

Vascular ultrasound enables detection of atherosclerotic plaques prior to calcification and can identify subclinical disease even in middle-aged, low-risk individuals [4, 5]. Moreover, ultrasound-based plaque burden assessment improves risk prediction beyond traditional clinical risk scores [6], with predictive performance comparable to that of coronary artery calcium (CAC) scoring, even when only carotid plaque burden is evaluated [7]. 

The presence of plaque alone has been shown to confer added prognostic value beyond conventional risk factors [8–10]. However, stratifying plaque burden beyond a binary presence/absence conveys additional information about degree of subclinical atherosclerosis and future CV risk [11, 12]. Still, models to quantify plaque burden have varied between studies [6, 13, 14], and there is a lack of consensus on which plaque burden model is the gold standard. Furthermore, although femoral plaque burden may provide incremental prognostic value, most prior studies have assessed carotid arteries alone. Evidence from large cohorts, such as the work-based PESA study, suggests that femoral plaque burden may exceed carotid burden [11]. In addition, femoral plaque burden has been shown to be more closely associated with coronary atherosclerosis [5] and to improve CV risk prediction beyond traditional risk factors and carotid plaque burden [15].

We recently developed and validated a novel plaque burden measure, that may be termed as weighted total plaque thickness (wTPT), which integrates plaque burden from carotid and femoral arteries across 12 vascular segments [16]. By capturing atherosclerosis across multiple vascular territories, this multiterritory approach may better reflect systemic atherosclerotic burden than carotid-only assessments. Moreover, we demonstrated that wTPT is strongly associated with CV risk factors, and correlates more closely with estimated CV risk, compared to traditional plaque burden measures [17]. In this study, we examined whether wTPT discriminates established CV disease more effectively than commonly used plaque measures. We also evaluated whether wTPT provides incremental discrimination beyond the NORRISK-2 algorithm and a conventional risk factor model. Finally, we compared the strength of association across increasing levels of global wTPT versus carotid MPT to assess whether a novel multiterritory measure captures atherosclerotic disease burden more accurately than a conventional carotid-based plaque metric.

## Materials and methods

**Fig. 1:**
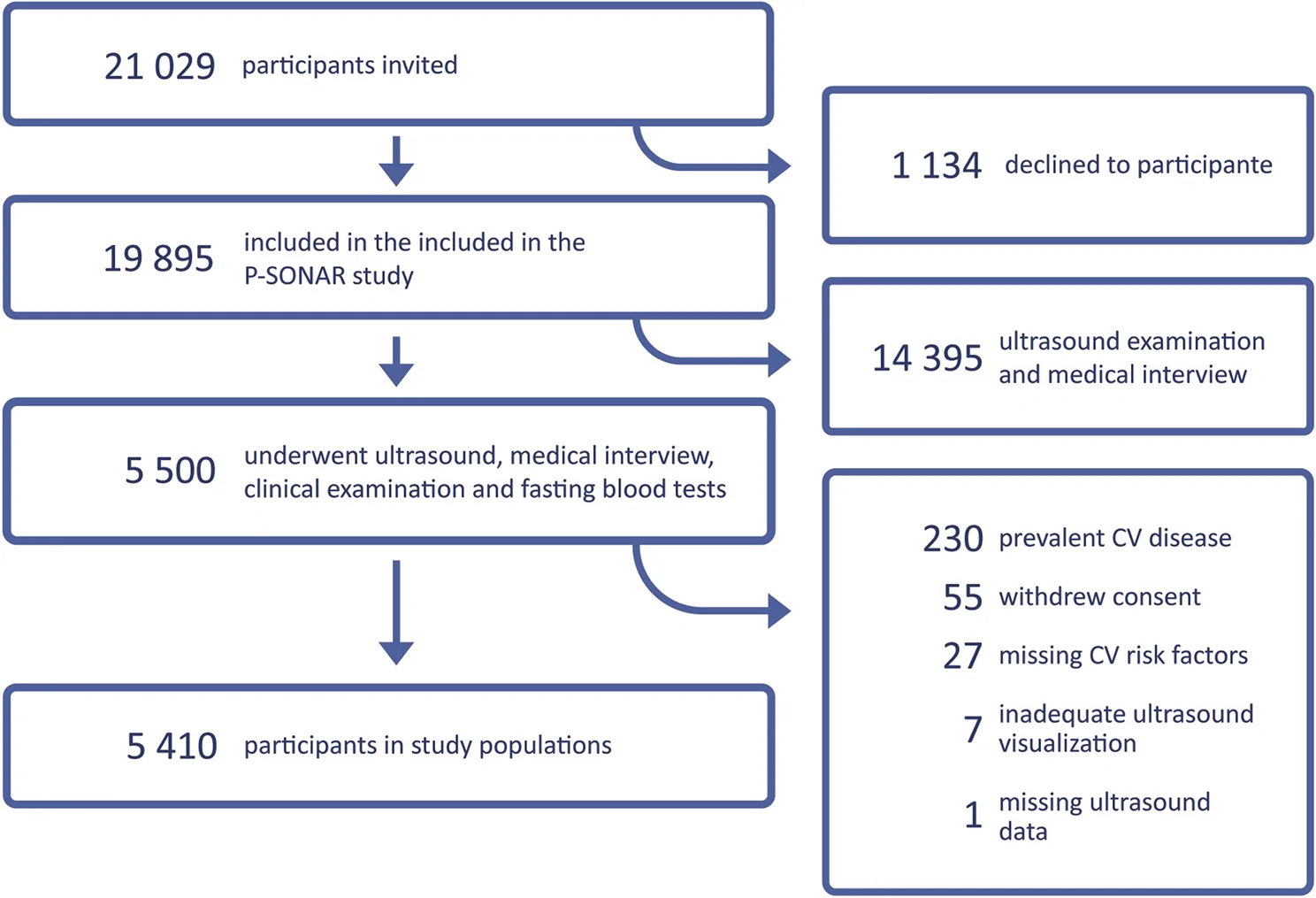
Flowchart of the Study Population

The Prospective Screening Of Non-invasive Atherosclerosis Risk (P-SONAR) is a prospective cohort study that has previously been described [17]. Briefly, between April 2022 to March 2025, 21 029 consecutive men and women 45–74 years who self-referred for an ultrasound-based health-check in one of 10 clinics in Norway were invited to participate. A total of 19 895 (94.6%) enrolled and underwent ultrasound examinations and medical interviews. In five clinics, a subgroup of 5 500 consecutive participants also provided fasting blood samples and underwent a clinical examination. Of the total, 55 withdrew consent, in 7 the ultrasound examination was inadequate, 1 had missing ultrasound data and 27 had missing information about CV risk factors, leaving 5 410 participants as the study sample (Fig. 1). The study protocol was approved by Regional Committees for Medical and Health Research Ethics in Norway (ref. number 258985). In accordance with the WORLD Medical Association Declaration of Helsinki, all participants provided written informed consent before entering the study [18]. 

The methodology of the 2D ultrasound examination and quantification of wTPT plaque burden have been previously reported [16]. Inter- and intra-observer reproducibility of wTPT measurements was evaluated in a dedicated validation study using repeated ultrasound examinations rather than repeated measurements on stored images, demonstrating high reproducibility across observers [16]. In brief, radiologists examined the carotid arteries (common carotid artery, carotid bifurcation, internal carotid artery) and the femoral arteries (common femoral artery, femoral bifurcation, superficial femoral artery). Plaque burden was quantified as wTPT, maximal plaque thickness (MPT), number of arteries with plaque and by plaque count. Plaque was defined according to the Mannheim Consensus Criteria [19], which define plaque as a focal structure encroaching into the arterial lumen of at least 0.5 mm or 50% of the surrounding intima–media thickness, or demonstrating a thickness > 1.5 mm. In the present study, only plaques ≥ 1.1 mm were registered to ensure inclusion of true atherosclerotic lesions and to avoid misclassification of intima–media thickening or measurement variability.

Prior CV disease was assessed through a medical interview conducted by the radiologists and included history of ischemic stroke, ischemic heart disease (IHD; including myocardial infarction and coronary revascularization) and peripheral artery disease (undergone peripheral arterial revascularization procedure). Levels of glucose, triglycerides, total cholesterol, LDL-C, HDL-C and Lipoprotein(a) (Lp(a)) were assessed through venous blood samples. Blood pressure and abdominal obesity were registered through clinical examination.

Estimated CV risk was calculated using the NORRISK-2 algorithm, which is a version of SCORE that has been validated for the Norwegian population [20]. The algorithm includes age, sex, current smoking, systolic blood pressure, total cholesterol, first-degree relatives with CV disease younger than 60 years, use of antihypertensive medication and low HDL-C. Additionally, we used a risk factor model to estimate CV risk, including sex, age, dyslipidemia, elevated Lp(a), diabetes, smoking history (yes/no), abdominal obesity and family history. Diagnosis of diabetes type 1 and type 2 was based on the medical interview. Participants (*n* = 42) with glucose ≥ 7.0 mmol/L (≥ 126 mg/dL) but no known diabetes were not classified as diabetic, as confirmatory testing was unavailable. Dyslipidemia was defined as total cholesterol > 6.2 mmol/l (> 240 mg/dL), LDL-C > 4.1 mmol/l (> 160 mg/dL) or HDL-C < 1.0 mmol/l (< 40 mg/dL), triglycerides ≥ 1.7 mmol/l (≥ 150 mg/dL) or use of cholesterol-lowering therapy. Elevated Lp(a) was defined as > 500 mg/L (> 50 mg/dL). Abdominal obesity was defined as waist circumference > 102 cm for men and > 88 cm for women. Blood pressure was measured after 5 min of rest; the mean of the second and third readings was used. Hypertension was defined as systolic BP > 140 mmHg, diastolic BP > 90 mmHg, or antihypertensive use.

The study population was stratified by the presence of prior CV disease. Continuous variables were listed as mean and standard deviation (SD) or as median and interquartile range (IQR). Categorical variables were presented as number and percentage or as percentage and 95% confidence interval (CI). Differences between continuous variables were tested by Student t-tests, whereas categorical data were tested by chi-square tests.

Logistic regression was used to estimate crude and adjusted odds ratios (OR) for CV disease by quartiles of plaque burden. The multivariable models were adjusted for the variables in the risk factor model.

Discrimination was assessed using c-statistic for NORRISK-2, the risk factor model and for each plaque burden model. Comparisons between c-statistics were performed using DeLong’s test for correlated receiver operating characteristic curves. Incremental performance of plaque burden was evaluated using the c-statistic, the net reclassification improvement (NRI), the integrated discrimination improvement (IDI) and the likelihood ratio test. To determine the best prognostic cutoff value for wTPT, we calculated the Youden J statistics from the c-statistic models. Participants with missing data on ultrasound measurements or CV risk factors were excluded from the analyses, and all statistical analyses were therefore performed using a complete case approach. Given the very low proportion of missing data, no imputation procedures were applied, and all analyses were conducted using complete cases. Statistical analyses were performed using Stata version 18.0. Level of significance of *p* < 0.05 was set for all analyses.

## Results

### Clinical characteristics

Clinical characteristics are presented in Table 1. Mean age was 59.8 ± 8.1 years and 2 866 (53.0%) were female. A total of 230 individuals (4.3%) had prior CV disease, including 165 (3.0%) with IHD, 55 (1.0%) with ischemic stroke and 10 (0.2%) with peripheral artery disease.

**Table 1:** Characteristics of the Study Population

	Total *N* = 5 418	Prior CV disease		*p* Value
		Yes *N* = 230	No *N* = 5 188	
Age, years	59.8 ± 8.1	65.2 ± 7.2	59.4 ± 8.1	< 0.001
Male	2548 (47.0)	168 (73.0)	2380 (45.9)	< 0.001
CV risk factors				
Dyslipidemia	3213 (59.3)	214 (93.0)	2999 (57.8)	< 0.001
Elevated Lp(a)	1150 (21.2)	69 (30.0)	1081 (20.8)	0.001
Diabetes	237 (4.4)	33 (14.4)	204 (3.9)	< 0.001
Hypertension	2 426 (44.8)	181 (78.7)	2245 (43.3)	< 0.001
Active smokers	527 (9.7)	24 (10.4)	414 (8.0)	0.181
Smoking history	1945 (38.4)	135 (58.7)	1945 (37.5))	< 0.001
Abdominal obesity	2371 (43.8)	103 (44.8)	2268 (43.7)	0.409
Family history				< 0.001
− 0	4504 (83.1)	148 (64.4)	4356 (84.0)	
− 1	817 (15.1)	72 (31.3)	745 (14.4)	
− 2+	97 (1.8)	10 (4.4)	87 (1.7)	
CV risk treatment				
Lipid lowering	1118 (20.6)	206 (89.6)	912 (17.6)	< 0.001
Antihypertensive	1221 (22.5)	148 (64.4)	1073 (20.7)	< 0.001
Blood thinning	576 (10.6)	216 (93.9)	360 (6.9)	< 0.001
CV risk				
NORRISK-2	5.8 (2.6–10.5)	12.0 (6.9–17.8)	5.6 (2.6–10.2)	< 0.001

*CV* cardiovascular, *Lp(a)* lipoprotein a, *Smoking history* current and/or prior smokers

Compared with individuals without prior CV disease, those with established CV disease had a higher prevalence of all cardiovascular risk factors, excepting abdominal obesity and current smoking. Moreover, a greater proportion of participants with established CV disease were past or current smokers (58.7% vs. 38.4%, *p* < 0.001).

### Association between plaque burden and prior CV disease

**Fig. 2:**
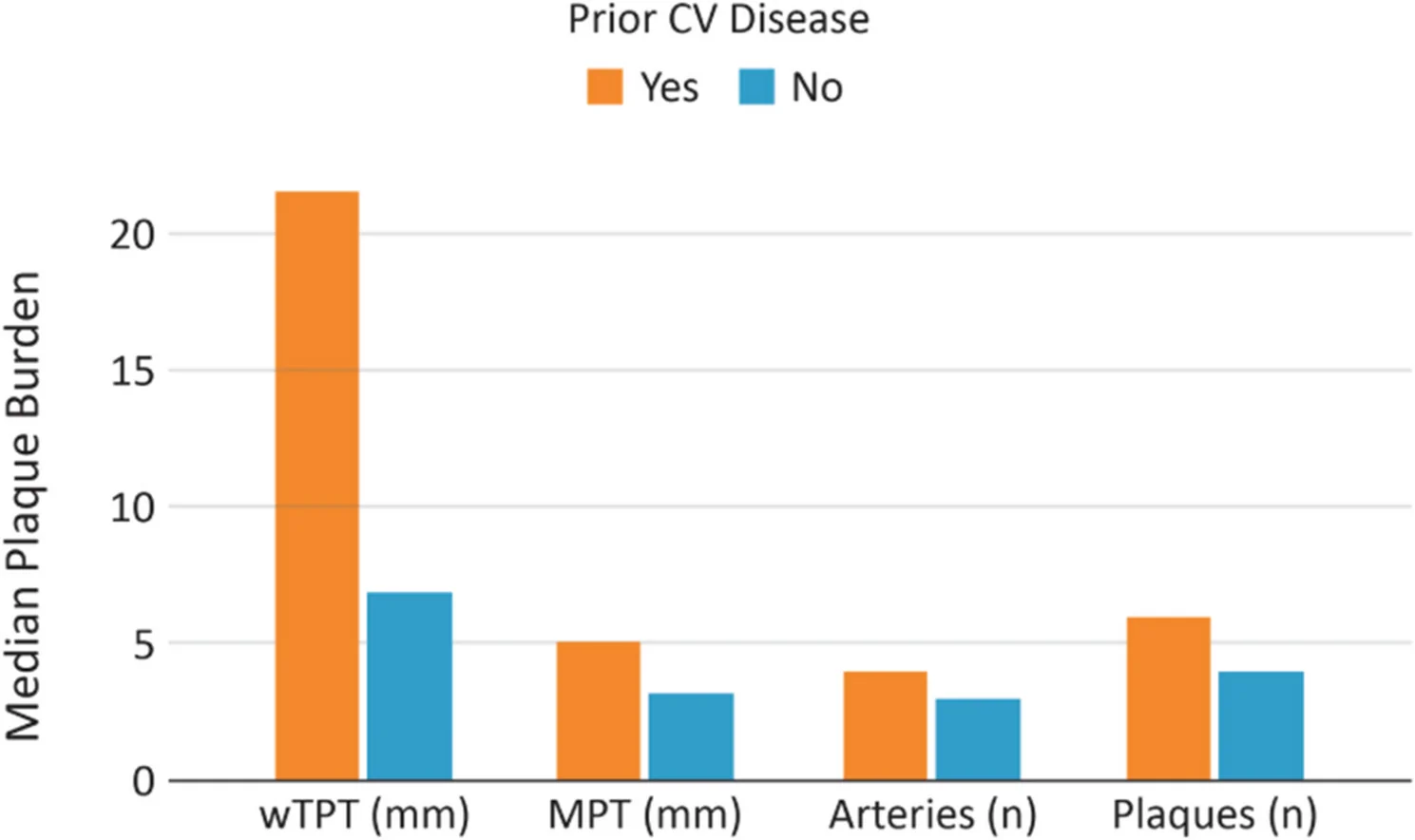
Plaque Burden by CV Disease. Bar graph illustrating median plaque burden across prior CV disease statusCV = cardiovascular. MPT = maximal plaque thickness. wTPT = weighted total plaque thickness

Overall, plaque prevalence was 99.1% in individuals with prior CV disease and 96.1% in those without (Table 2). Across all plaque burden measures, atherosclerosis was consistently higher in participants with established CV disease (Fig. 2). For wTPT, plaque burden was 21.5 mm (95% CI: 13.5–29.4) and 6.9 mm (95% CI: 3.6–12.4) in participants with and without prior CV disease, respectively.

**Table 2:** Plaque Burden by CV Disease

	Total *N* = 5 412	Prior CV disease	
		Yes *N* = 237	No *N* = 5 175
Plaque prevalence	5 212 (96.2)	228 (99.1)	4 984 (96.0)
carotid	4 907 (90.6)	226 (98.3)	4 681 (90.2)
femoral	4 363 (80.5)	225 (97.8)	4 138 (79.8)
Plaque count, IQR	4 (2–5)	6 (5–7)	4 (2–5)
Plaque count			
1–2	22.4 (20.5–24.4)	1.05 (0.03–5.7)	23.4 (21.6–25.6)
3	15.7 (14.1–17.5)	4.2 (1.2–10.4)	16.3 (14.6–18.1)
4–5	33.9 (31.7–36.0)	38.9 (29.1–49.5)	33.6 (31.4–35.8)
≥ 6	24.3 (22.4–26.3)	54.7 (44.2–65.0)	22.7 (20.7–24.7)
Arteries, IQR	3 (2–4)	4 (4–4)	3 (2–4)
Arteries			
1	8.1 (7.4–8.9)	0.43 (0.01–2.3)	8.4 (7.7–9.2)
2	19.3 (18.2–20.3)	2.2 (0.7-5.0)	20.0 (18.9–21.1)
3	20.6 (20.0-21.7)	6.5 (3.7–10.5)	21.2 (20.1–22.4)
4	48.2 (46.9–50.0)	90.0 (85-4-93.6)	46.4 (45.0-47.8)
MPT	3.3 (2.3–4.3)	5.1 (4.2–6.2)	3.2 (2.2–4.2)
wTPT	7.3 (3.6–13.2)	21.5 (13.5–29.4)	6.9 (3.6–12.4)

Categorical variables are presented as number and percentages. Plaque count and arteries with plaque are listed by percentages with 95% confidence interval. Non-normal distributed variables (Plaque count, Arteries, MPT, wTPT) are presented as median and interquartile range

*CV* cardiovascular, *IQR* interquartile range, *MPT* maximal plaque thickness, *wTPT* weighted total plaque thickness

In multivariate analyses, associations between prior CV disease and plaque burden were consistent across all models (Supplemental Table 1). In regards to wTPT, ORs were 2.5 (95% CI: 2.0-3.2), 1.6 (95% CI: 1.4–1.9) and 2.0 (95% CI: 1.7–2.5) for global, carotid and femoral plaque burden, respectively. Similar associations were observed for IHD and ischemic stroke for all individuals and when stratified by sex (Supplemental Tables 2–3). Compared to participants with plaque who were in the lowest global wTPT quartile for their 5-year age group and sex, the odds of having prior CV disease were 2.0 (0.89–4.4), 4.2 (2.0-8.6) and 10.0 (5.0-20.1) for those in quartiles 2, 3 and 4, respectively (Supplemental Tables 4 and Graphical abstract). Corresponding results for the established measure carotid MPT were 1.3 (95% CI: 0.73–2.3), 1.7 (95% CI: 0.99–2.9) and 3.4 (95% CI 2.1–5.7), respectively.

### Plaque burden, risk factors and prior CV disease

The c-statistic for discriminating prior CV disease was 0.72 (95% CI: 0.71–0.74) for arteries with plaque, 0.75 (95% CI: 0.71–0.79) for plaque count, 0.83 (95% CI: 0.80–0.86) for MPT, and 0.85 (95% CI: 0.83–0.87) for wTPT. wTPT significantly outperformed all other plaque burden models (Table 3). wTPT also demonstrated superior performance compared to the other plaque burden measures in identifying individuals with prior IHD and ischemic stroke, except for stroke, where the difference between wTPT (0.78 [95% CI: 0.72–0.84]) and MPT (0.76 [95% CI: 0.69–0.82]) did not reach statistical significance (*p* = 0.06).

**Table 3:** wTPT Discriminating Prior CV Disease Compared to Other Plaque Burden Measures

Variable	c-statistic (95% CI)	*p* Value
CV Disease		
Arteries	0.72 (0.71–0.74)	< 0.001
Plaque count	0.75 (0.71–0.79)	< 0.001
MPT	0.83 (0.80–0.86)	< 0.001
wTPT	0.85 (0.83–0.87)	NA
IHD		
Arteries	0.73 (0.71–0.75)	< 0.001
Plaque count	0.75 (0.71–0.79)	< 0.001
MPT	0.83 (0.81–0.87)	0.0038
wTPT	0.86 (0.83–0.88)	NA
IS		
Arteries	0.68 (0.63–0.73)	< 0.001
Plaque count	0,67 (0.59–0.76)	< 0.001
MPT	0.75 (0.69–0.82)	0.060
wTPT	0.78 (0.72–0.84)	NA

*p*-Value compared with weighted Total Plaque Thickness (wTPT)

*CV* cardiovascular, *IHD* ischemic heart disease, *IS* ischemic stroke, *MPT* maximal plaque thickness, *wTPT* weighted total plaque thickness

**Fig. 3:**
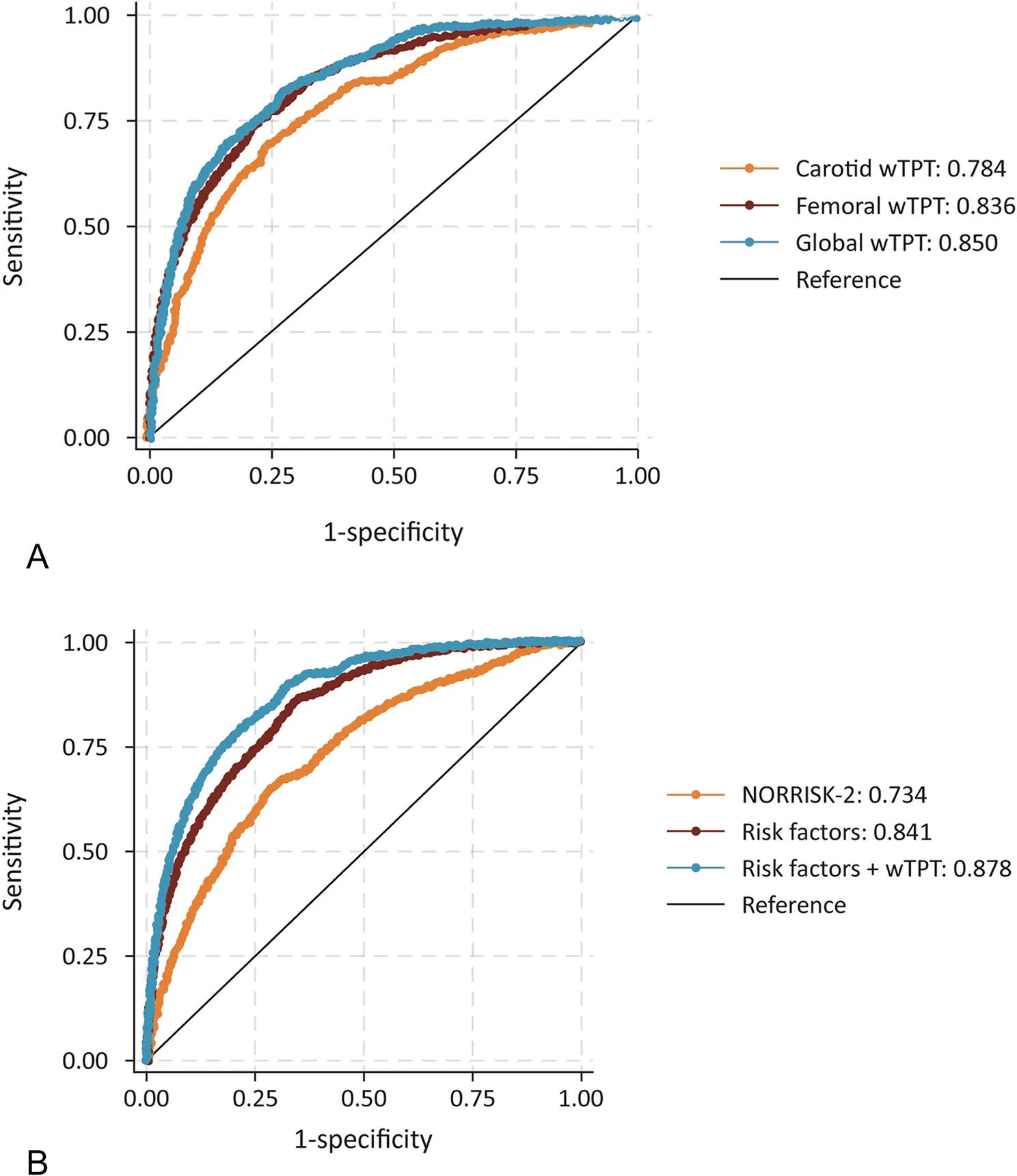
Receiver Operating Characteristics Curves for Discriminating Prior CV Disease. Receiver operating characteristic (ROC) curves for discrimination of prior cardiovascular disease. Figure **A**: ROC curves for carotid, femoral, and global wTPT plaque burden. Figure **B**: ROC curves for NORRISK-2, risk factor model, and risk factor model combined with global wTPT. Risk factors include sex, age, dyslipidemia, elevated lipoprotein a, diabetes, smoking history (yes/no), abdominal obesity and family history. CV = cardiovascular. RF = risk factors. wTPT = weighted total plaque thickness

Global wTPT yielded a higher c-statistic than femoral wTPT (0.84 [95% CI: 0.81–0.86]; *p* = 0.0142), which in turn outperformed carotid wTPT (0.78 [95% CI: 0.75–0.81]; *p* < 0.001) (Table 4, Fig. 3). The c-statistic for the NORRISK-2 score and for the risk factor model were 0.73 (95% CI: 0.71–0.77) and 0.84 (95% CI: 0.82–0.87), respectively. Adding global, femoral, or carotid wTPT to the risk factor model significantly improved discrimination; *p* < 0.001 for all. The combination of the risk factor model and global wTPT achieved a c-statistic of 0.88 (95% CI: 0.86–0.90), with a continuous NRI of 0.71 (95% CI: 0.57–0.84) and IDI of 0.06 (95% CI: 0.04–0.07) (Fig. 3). Similar improvements were observed for prior IHD and ischemic stroke (Supplemental Table 5), though for stroke, the added value of carotid wTPT did not reach statistical significance (*p* = 0.079). Comparable improvements were also observed when stratified by sex, however, increase in c-statistic for IHD in females, as well as ischemic stroke for both sexes, did not reach statistical significance (Supplemental Table 6).

**Table 4:** Incremental Value of wTPT to Risk Factors in Discriminating CV Disease

Variable	c-statistic (95% CI)	*p* Value*	Continuous NRI (95% CI)	IDI (95% CI)	Test statistics	*p* Value**
NORRISK-2	0.73 (0.71–0.77)	N/A	N/A	N/A	N/A	N/A
RF	0.84 (0.82–0.87)	Ref.	Ref.	Ref.	Ref.	Ref.
wTPT global	0.85 (0.83–0.87)	N/A	N/A	N/A	N/A	N/A
wTPT + RF	0.88 (0.86–0.90)	< 0.001	0.71 (0.57–0.84)	0.06 (0.04–0.07)	122.6	< 0.001
wTPT carotid	0.78 (0.75–0.81)	N/A	N/A	N/A	N/A	N/A
wTPT carotid + RF	0.86 (0.84–0.88)	< 0.001	0.39 (0.25–0.52)	0.02 (0.01–0.03)	44.8	< 0.001
wTPT femoral	0.84 (0.81–0.86)	N/A	N/A	N/A	N/A	N/A
wTPT femoral + RF	0.87 (0.85–0.90)	< 0.001	0.67 (0.53–0.80)	0.05 (0.04–0.06)	114.9	< 0.001

*CV* cardiovascular, *IDI* integrated discrimination improvement, *NRI* net reclassification improvement, *IDI* integrated discrimination improvement, *RF* risk factors, including sex, age, dyslipidemia, hypertension, past or current smoking, diabetes, family history and elevated lipoprotein a, *Test statistics* likelihood ratio test statistics, *wTPT* weighted Total Plaque Thickness

*p value compared with risk factors

**p Value Likelihood ratio test

### Prognostic cutoff value for wTPT plaque burden

Using the Youden J statistic, a wTPT threshold of > 11.4 mm was identified as the optimal cut point for discriminating established CV disease, yielding a sensitivity of 83.5%, specificity of 71.0%, positive predictive value of 11.3%, and a negative predictive value of 99.0%. This cut-off was derived for statistical discrimination in this study context and not intended as a clinical decision threshold. The odds of having prior CV disease in participants above this threshold were 4.6 (95% CI: 3.1–6.9). When the wTPT cutoff was added to the risk factor model, the c-statistic increased to 0.87 (95% CI 0.85–0.89). Furthermore, the continuous NRI was 0.73 (0.61–0.86) and the IDI was 0.017 (95% CI: 0.012–0.021).

## Discussion

Our findings demonstrate that wTPT, a novel method based on the weighted sum of plaque thickness across 12 vascular segments in carotid and femoral arteries, is superior to conventional models to measure plaque burden in discriminating between individuals with prior CV disease versus those without prior disease. Furthermore, wTPT added substantial incremental discriminative value beyond standard risk factors, as reflected by an improved c-statistic and a marked increase in NRI. Notably, global wTPT provided superior discrimination compared to femoral or carotid wTPT alone, with assessments of femoral burden outperforming that of carotid burden. Additionally, individuals with high wTPT plaque burden had substantially elevated odds of prior CV disease compared with those classified by the conventional carotid MPT measure.

### Assessing subclinical atherosclerosis by ultrasound

Studies of plaque prevalence in low to medium risk individuals have varied substantially, particularly for carotid plaques [21, 22]. However, with modern ultrasound technology and experienced sonographers, high prevalence rates of carotid plaque have been reported [22]. In our study, plaque was present in 95.8% of participants without prior CV disease, indicating that a simple binary classification of plaque presence offers limited discriminatory value for risk prediction.

Plaque volume assessed by 3D ultrasound may represent the most comprehensive measure of subclinical atherosclerosis, incorporating plaque number, height, width, and length [11]. However, this method is time-consuming and impractical for routine clinical use [16]. Furthermore, the BioImage study showed that carotid maximal plaque thickness (MPT) had comparable predictive utility to total plaque volume (TPV) [13]. wTPT serves as a surrogate for TPV, integrating plaque number, thickness, and length, while being more time-efficient to acquire [16]. Since wTPT relies on detailed 2D scanning of all arterial segments, it may also detect a broader range of subclinical disease compared to standardised 3D sweeps [16]. We have previously shown that wTPT correlates more strongly with estimated CV risk than MPT, plaque count, or number of affected arteries [17]. Consistently, in this study, wTPT outperformed all other plaque burden measures in discriminating prior CV disease. Adding wTPT to both NORRISK-2 and the traditional risk factor model significantly improved the c-statistic and NRI, supporting wTPT as a practical and predictive tool for assessing atherosclerotic burden in clinical settings.

### Femoral plaque burden

Femoral plaque burden has been shown to be more extensive than carotid plaque burden [11] and more closely correlated with coronary artery calcium (CAC) scores [5]. Additionally, it improves CV risk prediction when added to traditional risk factors and carotid plaque burden [15]. Nevertheless, most ultrasound-based studies have focused solely on the carotid arteries.

Recently, we demonstrated that femoral plaque burden exceeded carotid burden, and that global wTPT correlated most strongly with estimated CV risk, with the femoral territory contributing more than the carotid territory [17]. In the present study, global wTPT had the highest discriminatory power for prior CV disease, followed by femoral and then carotid wTPT. These findings were consistent across sexes. The superior performance of femoral over carotid wTPT reinforces the importance of including femoral arteries when evaluating subclinical atherosclerosis by ultrasound for CV risk assessment.

### Discrimination of IHD and ischemic stroke

The ACE1950 study found that carotid plaque burden was more strongly associated with incident ischemic stroke than with MACE, likely due to the role of carotid plaques as a potential thromboembolic source [6]. However, in the MESA study, the association seemed to be higher between carotid plaque presence and both CV disease and coronary heart disease, compared to ischemic stroke or transient ischemic attack [14]. In the present study, predictive performance of both NORRISK-2, the risk factor model and all plaque burden models seemed to be lower for ischemic stroke, compared to both IHD and CV disease. As only a minority of ischemic strokes are caused by large vessel atherosclerosis, whereas most IHD are caused by rupture of coronary artery plaques, these findings might not be too surprising [23, 24]. 

### Study limitations

For measures of plaque burden to yield clinically meaningful information, they must improve prediction beyond traditional risk models. However, conventional risk scores like NORRISK-2 are not designed to discriminate prior CV disease. Participants with established disease often initiate lifestyle changes and medical therapy, potentially altering their current risk profile. Indeed, as expected, use of lipid-lowering, antihypertensive, antidiabetic, and antithrombotic medications was significantly higher in those with established CV disease in our study. While the proportion of current smokers was similar between groups, former smoking was more common among those with prior disease, and no significant difference in abdominal obesity was observed. Given these factors, it is unsurprising that NORRISK-2 demonstrated only modest discriminatory ability (c-statistic 0.73; 95% CI:0.71–0.77). To better reflect underlying risk, we evaluated an alternative model incorporating age, sex, dyslipidemia, hypertension, diabetes, elevated Lp(a), past or current smoking, and family history. In this context, where 90% of participants with prior CV disease were on lipid-lowering therapy, dyslipidemia likely provided stronger risk stratification than total cholesterol, which is used in NORRISK-2. This risk factor model achieved a relatively high c-statistic of 0.84 (95% CI: 0.82–0.87), supporting the clinical value of the observed improvements in discrimination and NRI when adding wTPT. Additionally, as lipid lowering therapy reduces the progression of subclinical atherosclerosis and may in fact reduce plaque volume [25], the added value of wTPT for identifying prior CV disease might be underestimated. Moreover, the multivariate ORs for prior CV disease observed in the highest carotid MPT quartile in our study were very similar to the associations observed between carotid MPT and incident CV disease in recent studies [13]. 

In the present study, radiologists were not blinded to participants’ risk factors or history of prior CV disease during ultrasound examinations. This introduces the potential for observer bias, possibly leading to higher plaque burden registration in participants with established disease. However, the high plaque prevalence even among participants without prior CV disease suggests that any such bias was likely minimal. Moreover, it is unlikely that this limitation significantly influenced the comparative performance of different plaque burden measures or vascular territories.

Although wTPT demonstrated strong discriminatory performance for established CV disease, this cross-sectional analysis cannot be directly extrapolated to risk prediction. Prospective validation with incident CV outcomes is therefore required before application in risk prediction or clinical decision-making.

### Strengths of the study

Ultrasound examinations were conducted by radiologists with extensive experience in vascular imaging, using standardised equipment. Plaque assessment was comprehensive, covering 12 vascular segments across both carotid and femoral territories, and allowing for direct comparison of multiple plaque burden measures. Importantly, examinations were performed in routine clinical settings rather than specialised research environments, supporting the generalisability of our findings. The study included a large, diverse cohort spanning a wide age range and multiple regions of Norway. Finally, we applied a broad range of statistical measures to assess the relationship between plaque burden and prior CV disease, with consistent results supporting the superior performance of wTPT.

## Conclusion

Global wTPT showed superior discrimination of established CV disease compared to established plaque measures and added substantial incremental discriminative value beyond traditional risk factors. These findings support wTPT as a marker of systemic atherosclerosis and a strong candidate for future outcome-based risk refinement.

## Supplementary Information

Supplementary Material 1.

## Data Availability

Because of the sensitive nature of the data in this study, participants were assured that raw data would remain confidential and would not be shared.

## Abbreviations

P-SONAR: Prospective Screening Of Non-invasive Atherosclerosis Risk
CV: Cardiovascular
wTPT: Weighted total plaque thickness
MPT: Maximal plaque thickness
TPV: Total plaque volume
CAC: Coronary artery calcium
SCORE: Systematic Coronary Risk Evaluation
Lp(a): Lipoprotein a
IHD: Ischemic heart disease
