# Correction: Discriminating established cardiovascular disease using a novel multiterritory ultrasound plaque burden measure (wTPT): findings from the P-SONAR study

**DOI:** 10.1186/s12872-026-06711-x

**Published:** 2026-10-05

**Authors:** Gunnar Austad, Jonn Terje Geitung, Owen Thomas, Serena Tonstad

**Affiliations:** 1 Department of Preventive Cardiology, Oslo University Hospital, Oslo, N-0424 Norway; 2 Faculty of Medicine, University of Oslo, Oslo, Norway; 3 Department of Radiology and Nuclear Medicine, Akershus University Hospital, Lørenskog, N-1478 Norway; 4 Health Services Research Unit, Akershus University Hospital, Lørenskog, N-1478 Norway

**Correction: BMC Cardiovasc Disord 26, 428 (2026)**

**https://doi.org/10.1186/s12872-026-05807-8**

In this article [1], Fig(s) 1, 3 and Graphical Abstract appeared incorrectly and have now been corrected in the original publication. For completeness and transparency, the old incorrect versions are displayed below.

Incorrect Figures

Graphical Abstract

**Figure:**
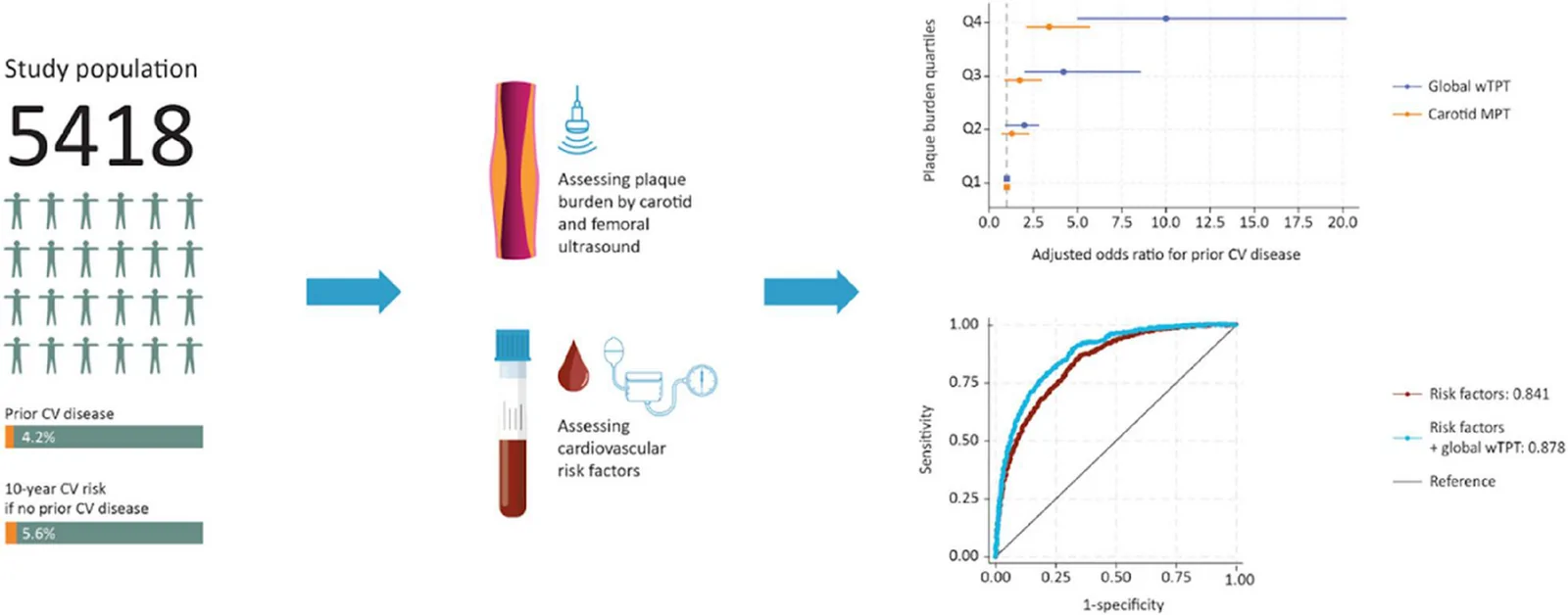

**Fig. 1:**
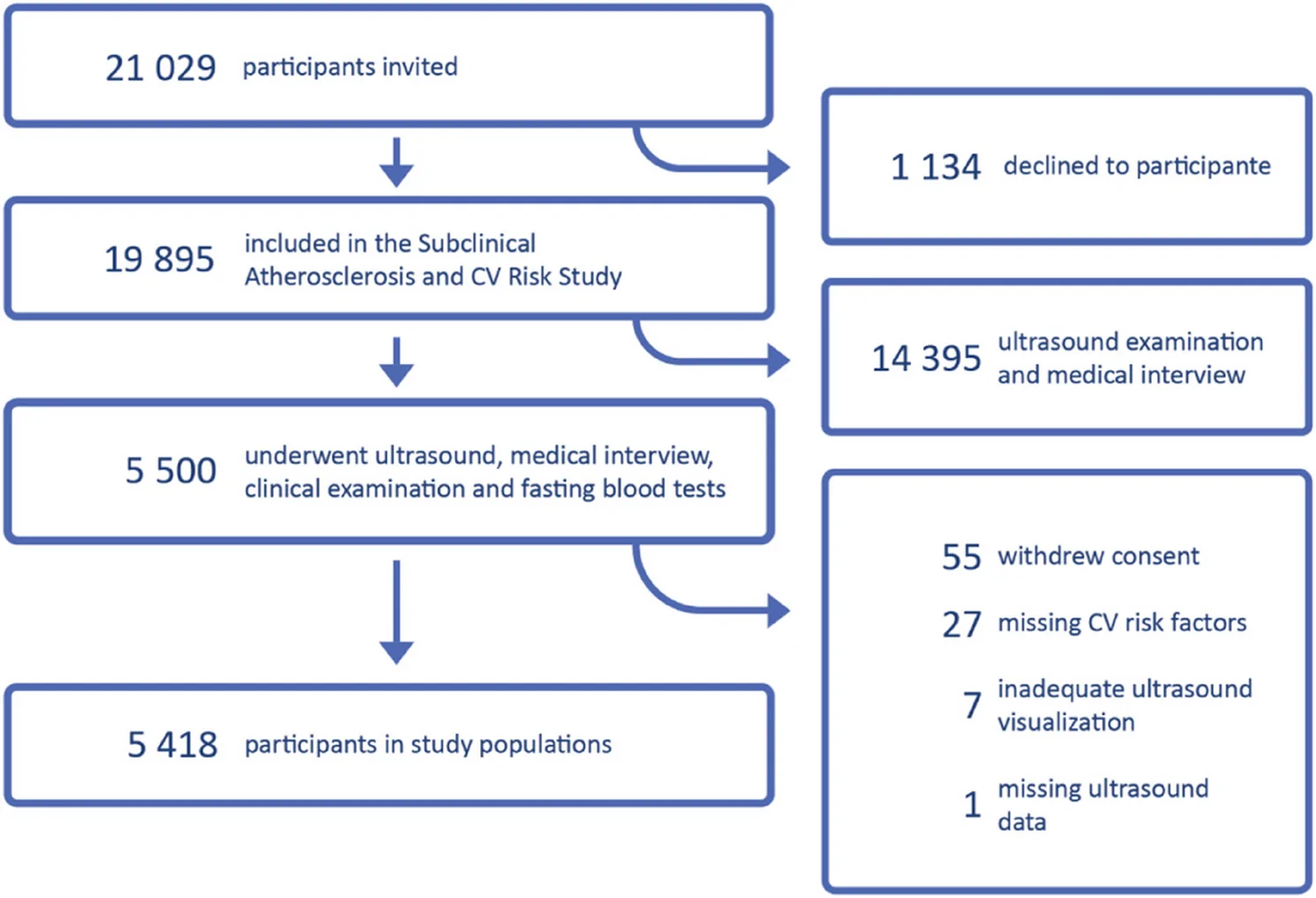
Flowchart of the Study Population

**Fig. 2:**
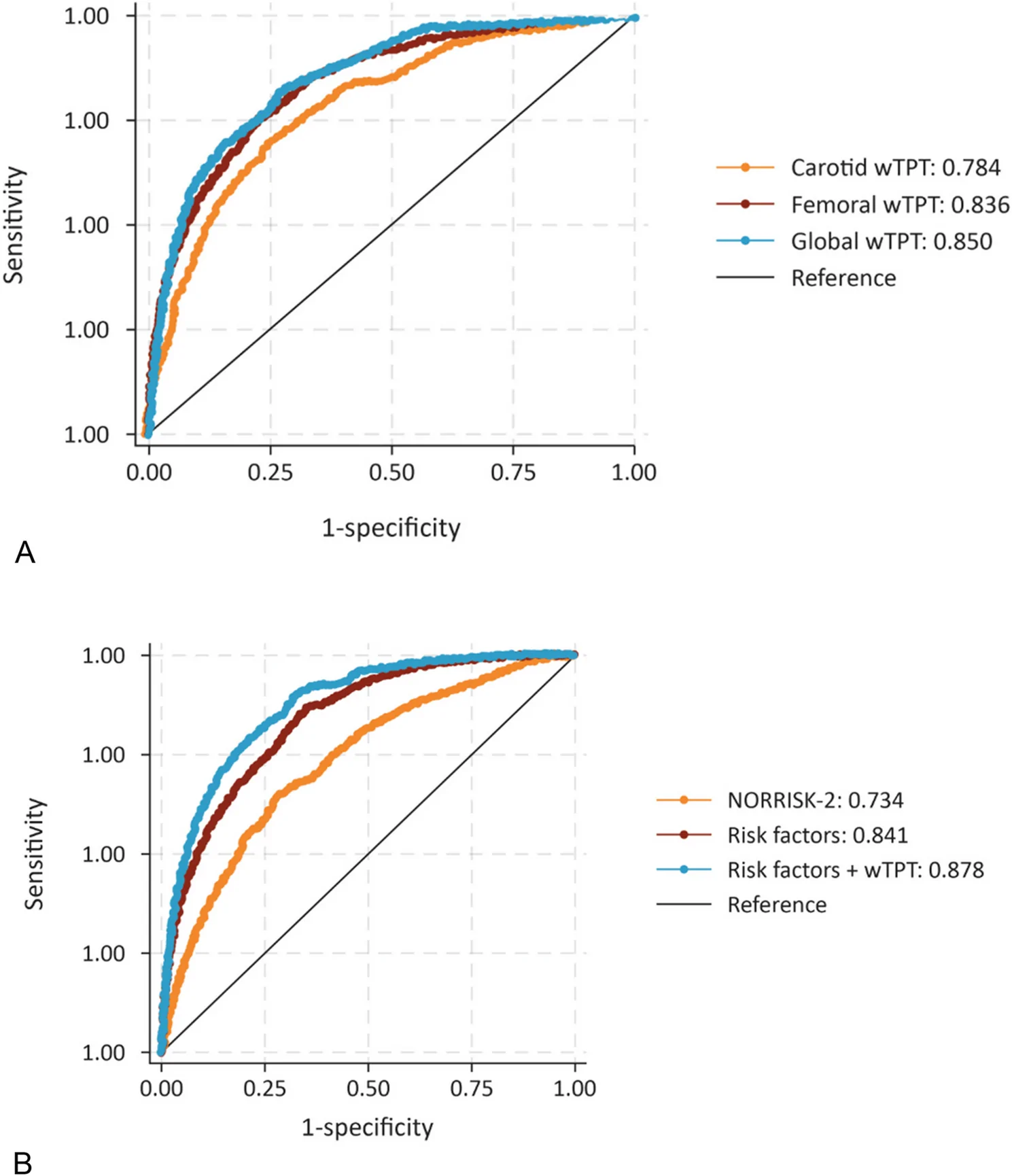
Plaque Burden by CV Disease. Bar graph illustrating median plaque burden across prior CV disease statusCV = cardiovascular. MPT = maximal plaque thickness. wTPT = weighted total plaque thickness

Corrected Figures

Graphical Abstract

**Figure:**
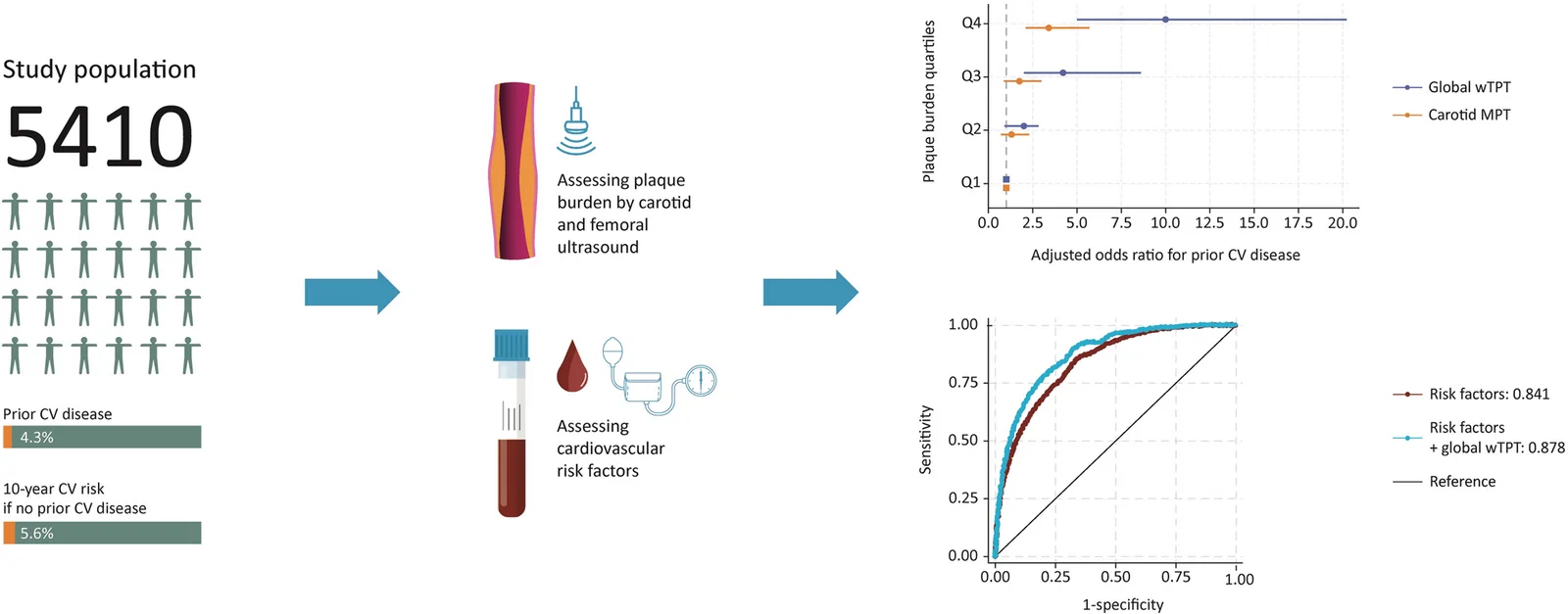

**Fig. 1:**
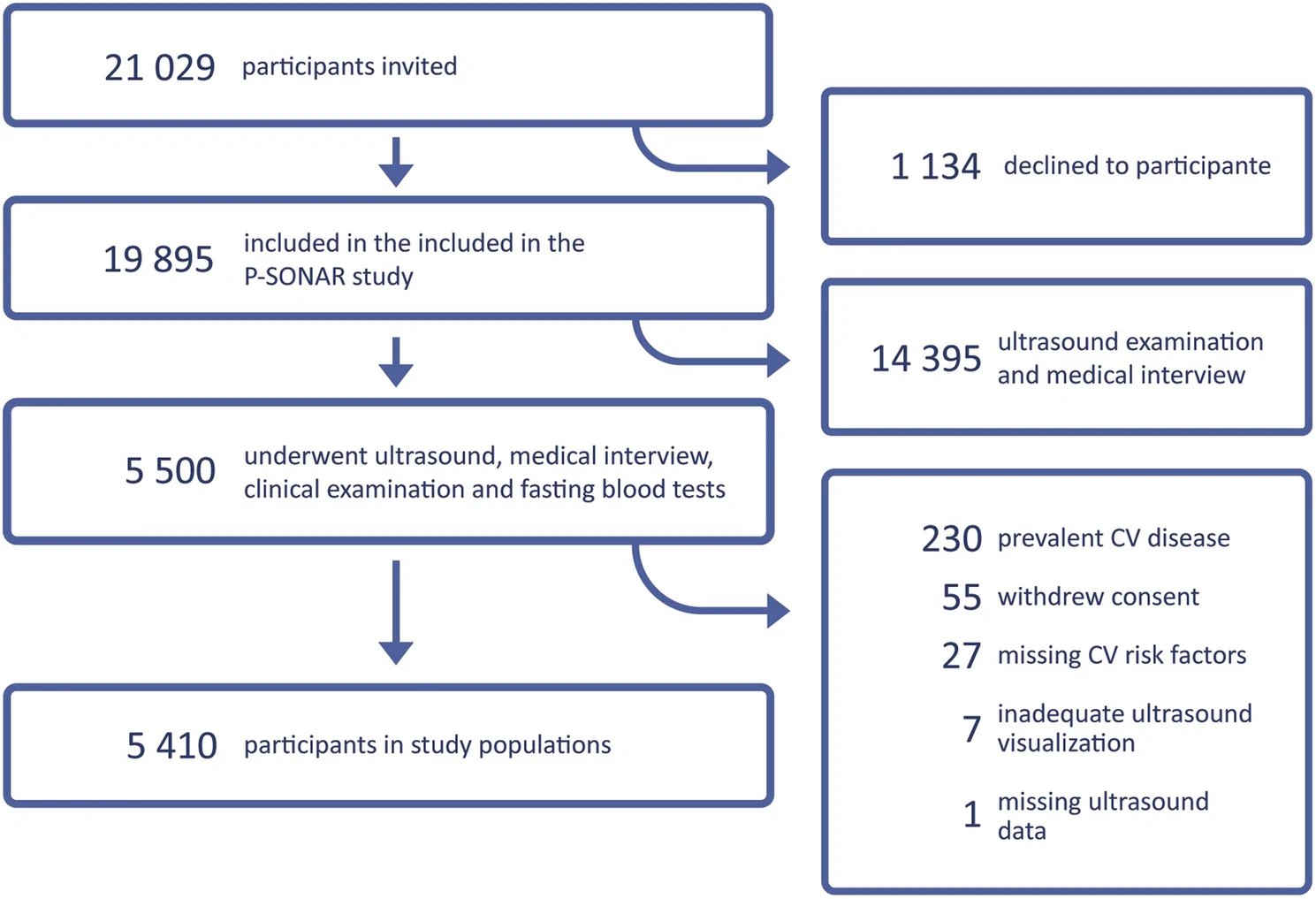
Flowchart of the Study Population

**Fig. 2:**
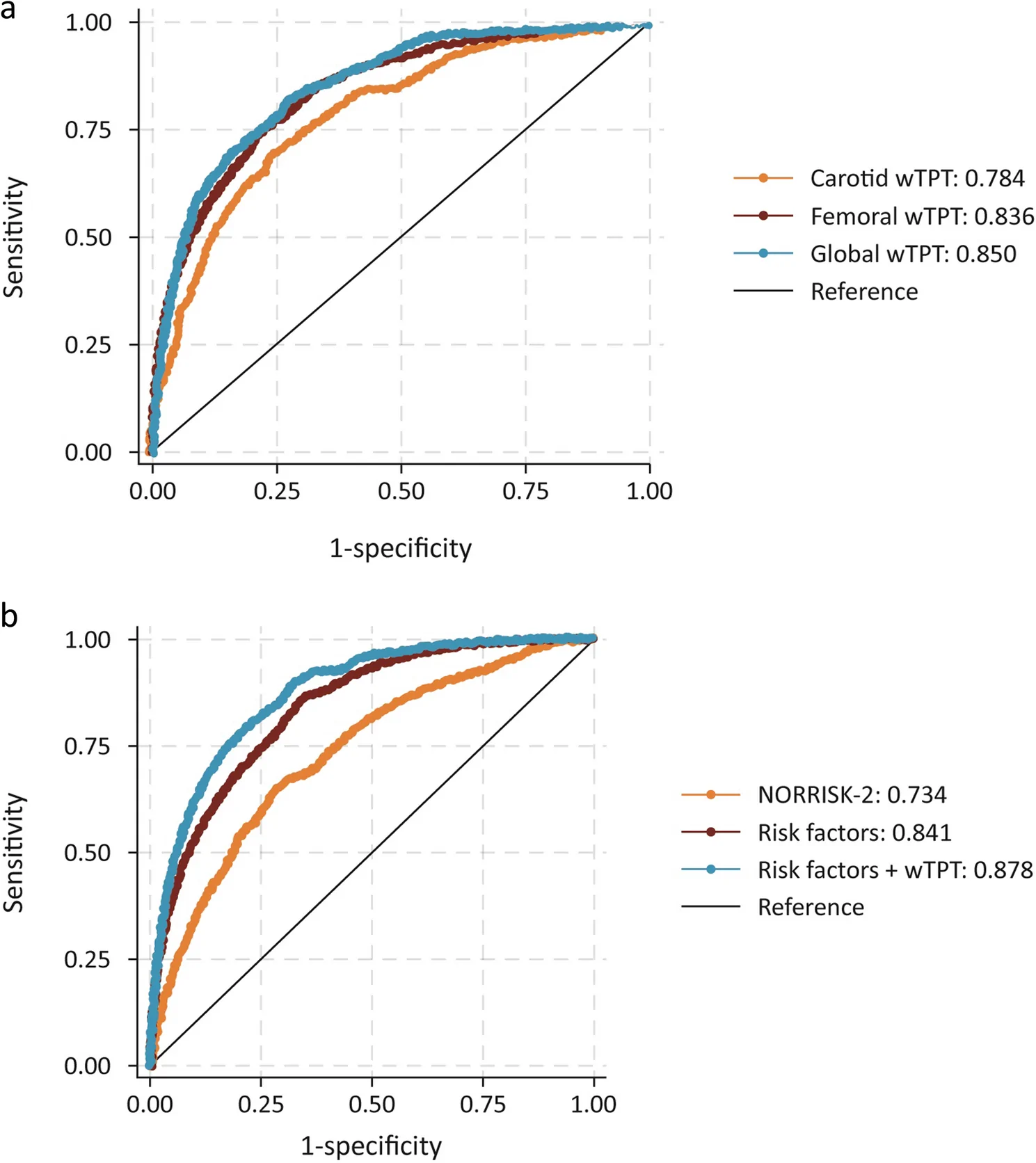
Plaque Burden by CV Disease. Bar graph illustrating median plaque burden across prior CV disease statusCV = cardiovascular. MPT = maximal plaque thickness. wTPT = weighted total plaque thickness
