# Supplementary material for: Discriminating established cardiovascular disease using a novel multiterritory ultrasound plaque burden measure (wTPT): findings from the P-SONAR study
Source: BMC Cardiovasc Disord. 2026 Apr 9;26:428. doi: 10.1186/s12872-026-05807-8 (PMC13202812; doi:10.1186/s12872-026-05807-8)
Supplement: Supplementary file 1 — Supplementary Material 1. [file 12872_2026_5807_MOESM1_ESM.docx]

Supplemental Material

## Supplemental Table 1. Univariate and Multivariate Plaque Burden Predictors of prior CV Disease

|  | **Univariate** | **p Value** | **Multivariate** | **p Value** |
| --- | --- | --- | --- | --- |
| Arteries | 4.8 (3.4-6.7) | < 0.001 | 2.5 (1.7-3.5) | < 0.001 |
| Plaques | 2.8 (2.1-3.8) | < 0.001 | 1.9 (1.4-2.6) | < 0.001 |
| MPT quartiles | 3.7 (3.0-4.5) | < 0.001 | 2.3 (1.8-2.9) | < 0.001 |
| - carotid MPT quartiles | 2.5 (2.1-2.9) | < 0.001 | 1.6 (1.4-1.9) | < 0.001 |
| - femoral MPT quartiles | 2.7 (2.3-3.2) | < 0.001 | 1.8 (1.6-2.2) | < 0.001 |
| wTPT quartiles | 4.0 (3.3-5.0) | < 0.001 | 2.5 (2.0-3.2) | < 0.001 |
| - carotid wTPT quartiles | 2.6 (2.2-3.0) | < 0.001 | 1.6 (1.4-1.9) | < 0.001 |
| - femoral wTPT quartiles | 3.1 (2.6-3.7) | < 0.001 | 2.0 (1.7-2.5) | < 0.001 |

Odds ratio for prior CV disease by plaque burden quartiles among participants with subclinical atherosclerosis. Multivariate analysis adjusted for age, sex, dyslipidemia, elevated Lp(a), diabetes, hypertension, past or current smoking, family history and abdominal obesity.

Arteries = Arteries with plaque (1-4). CV = cardiovascular. MPT = maximal plaque thickness. Plaques = 1-2, 3, 4-5, 6+. wTPT = weighted total plaque thickness.

## Supplemental Table 2. Univariate and Multivariate Plaque Burden Predictors of Prior IHD and Ischemic Stroke

|  | **Univariate** | **p Value** | **Multivariate** | **p Value** |
| --- | --- | --- | --- | --- |
| IHD |  |  |  |  |
| Arteries | 5.7 (3.7-9.0) | < 0.001 | 2.7 (1.7-4.3) | < 0.001 |
| Plaques | 2.7 (2.0-3.7) | < 0.001 | 1.7 (1.2-2.5) | 0.004 |
| MPT quartiles | 4.1 (3.2-5.2) | < 0.001 | 2.4 (1.8-3.1) | < 0.001 |
| - carotid MPT  quartiles | 2.5 (2.0-3.0) | < 0.001 | 1.5 (1.2-1.9) | < 0.001 |
| - femoral MPT  quartiles | 3.0 (2.4-3.6) | < 0.001 | 1.9 (1.5-2.4) | < 0.001 |
| wTPT quartiles | 4.3 (3.3-5.6) | < 0.001 | 2.4 (1.8-3.2) | < 0.001 |
| - carotid wTPT  quartiles | 2.6 (2.1-3.1) | < 0.001 | 1.5 (1.2-1.8) | < 0.001 |
| - femoral wTPT  quartiles | 3.4 (2.7-4.2) | < 0.001 | 2.1 (1.7-2.7) | < 0.001 |
| IS |  |  |  |  |
| Arteries | 2.9 (1.8-4.8) | < 0.001 | 1.8 (1.1-3.1) | 0.028 |
| Plaques | 2.8 (1.5-5.1) | 0.001 | 2.2 (1.1-4.5) | 0.025 |
| MPT quartiles | 2.4 (1.7-3.2) | < 0.001 | 1.7 (1.2-2.5) | 0.003 |
| - carotid MPT  quartiles | 2.1 (1.5-2.8) | < 0.001 | 1.6 (1.1-2.2) | 0.005 |
| - femoral MPT  quartiles | 1.9 (1.5-2.4) | < 0.001 | 1.5 (1.1-2.0) | 0.008 |
| wTPT quartiles | 4.3 (3.3-5.6) | < 0.001 | 2.2 (1.5-3.3) | < 0.001 |
| - carotid wTPT  quartiles | 2.6 (2.1-3.1) | < 0.001 | 1.6 (1.2-2.2) | 0.003 |
| - femoral wTPT  quartiles | 3.4 (2.7-4.2) | < 0.001 | 1.8 (1.3-2.4) | 0.001 |

Odds ratio for prior ischemic heart disease (IHD) and ischemic stroke (IS) by plaque burden quartiles among participants with subclinical atherosclerosis. Multivariate analysis adjusted for age, sex, dyslipidemia, elevated Lp(a), diabetes, hypertension, past or current smoking, family history and abdominal obesity

Arteries = Arteries with plaque (1-4). CV = cardiovascular. MPT = maximal plaque thickness. Plaques = 1-2, 3, 4-5, 6+. wTPT = weighted total plaque thickness.

## Supplemental Table 3. Univariate and Multivariate Plaque Burden Predictors of prior CV Disease by Sex

| **Plaque Burden** | **Sex** | **Univariate** | **p Value** | **Multivariate** | **p Value** |
| --- | --- | --- | --- | --- | --- |
| CV Disease |  |  |  |  |  |
| Arteries | Females | 3.1 (2.0-4.9) | < 0.001 | 2.1 (1.3-3.3) | 0.002 |
|  | Males | 6.1 (3.6-10.4) | < 0.001 | 3.0 (1.8-5.1) | < 0.001 |
| Plaques | Females | 2.7 (1.7-4.4) | < 0.001 | 1.8 (1.0-3.1) | 0.039 |
|  | Males | 2.6 (1.8-3.7) | < 0.001 | 1.8 (1.2-2.7) | 0.004 |
| MPT quartiles | Females | 3.0 (2.1-4.3) | < 0.001 | 2.1 (1.4-3.0) | < 0.001 |
|  | Males | 3.6 (2.9-4.5) | < 0.001 | 2.4 (1.9-3.1) | < 0.001 |
| wTPT quartiles | Females | 3.5 (2.4-5.1) | < 0.001 | 2.5 (1.7-3.8) | < 0.001 |
|  | Males | 4.0 (3.1-5.1) | < 0.001 | 2.7 (2.0-3.5) | < 0.001 |
| IHD |  |  |  |  |  |
| Arteries | Females | 3.7 (1.8-7.6) | < 0.001 | 2.6 (1.2-5.5) | 0.016 |
|  | Males | 5.9 (3.3-10.4) | < 0.001 | 2.9 (1.6-5.1) | < 0.001 |
| Plaques | Females | 2.0 (1.1-3.8) | 0.026 | 1.2 (0.5-2.5) | 0.716 |
|  | Males | 2.4 1.7-3.5) | < 0.001 | 1.7 (1.1-2.6) | 0.014 |
| MPT quartiles | Females | 3.7 (2.1-6.5) | < 0.001 | 2.4 (1.3-4.4) | 0.007 |
|  | Males | 3.6 (2.8-4.7) | < 0.001 | 2.4 (1.8-3.2) | < 0.001 |
| wTPT quartiles | Females | 4.0 (2.2-7.3) | < 0.001 | 2.8 (1.4-5.4) | 0.002 |
|  | Males | 3.9 (3.0-5.1) | < 0.001 | 2.6 (1.9-3.5) | < 0.001 |
| IS |  |  |  |  |  |
| Arteries | Females | 2.4 (1.4-4.2) | 0.002 | 1.5 (0.8-2.8) | 0.172 |
|  | Males | 5.5 (1.5-19.9) | 0.009 | 3.2 (0.9-11.7) | 0.079 |
| Plaques | Females | 2.9 (1.4-6.1) | 0.004 | 2.0 (0.9-4.5) | 0.106 |
|  | Males | 4.0 (1.0-15.4) | 0.047 | 2.9 (0.7-12.2) | 0.144 |
| MPT quartiles | Females | 2.4 (1.4-3.5) | < 0.001 | 1.5 (0.9-2.5) | 0.089 |
|  | Males | 2.6 (1.6-4.3) | < 0.001 | 1.9 (1.1-3.2) | 0.027 |
| wTPT quartiles | Females | 2.7 (1.7-4.3) | < 0.001 | 1.9 (1.1-3.2) | 0.021 |
|  | Males | 3.1 (1.8-5.1) | < 0.001 | 2.2 (1.2-4.1) | 0.008 |

Odds ratio for prior cardiovascular (CV) disease, ischemic heart disease (IHD) and ischemic stroke (IS) by plaque burden quartiles among participants with subclinical atherosclerosis, stratified by sex. Multivariate analysis adjusted for age, sex, dyslipidemia, elevated Lp(a), diabetes, hypertension, past or current smoking, family history and abdominal obesity

Arteries = Arteries with plaque (1-4). MPT = maximal plaque thickness. Plaques = 1-2, 3, 4-5, 6+. wTPT = weighted total plaque thickness.

## Supplemental Table 4. Odds Ratios for Established CV Disease associated with wTPT and MPT

|  | **Quartile 1** | **Quartile 2** | **Quartile 3** | **Quartile 4** |
| --- | --- | --- | --- | --- |
| **wTPT** |  |  |  |  |
| **- Global** | 1.0 (ref) | 2.0 (0.89-4.4) | 4.2 (2.0-8.6) | 10.0 (5.0-20.1) |
| **- Carotid** | 1.0 (ref) | 1.8 (1.0-3.1) | 2.2 (1.3-3,8) | 3.7 (2.2-6-2) |
| **- Femoral** | 1.0 (ref) | 1.3 (0.67-2.7) | 2.5 (1.4-4.6) | 6.1 (3.4-11.0) |
| **MPT** |  |  |  |  |
| **- Global** | 1.0 (ref) | 1.4 (0.67-3.0) | 3.0 (1.5-5.9) | 6.8 (3.6-13.0) |
| **- Carotid** | 1.0 (ref) | 1.3 (0.73-2.3) | 1.7 (0.99-2.9) | 3.4 (2.1-5.7) |
| **- Femoral** | 1.0 (ref) | 0.7 (0.38-1.29) | 1.3 (0.79-2.2) | 3.4 (2.1-5.5) |

Odds ratio for prior cardiovascular disease associated with weighted total plaque thickness (wTPT) and maximal plaque thickness (MPT) in carotid arteries, femoral arteries and in combined carotid and femoral (global) arteries. Odds ratios were adjusted for age, sex, dyslipidemia, elevated Lp(a), diabetes, hypertension, past or current smoking and family history.

## Supplemental Table 5. Incremental Value of wTPT to Risk Factors in Discriminating IHD and Ischemic Stroke

| **Variable** | **c-statistic (95% CI)** | **p Value*** | **Continuous NRI (95% CI)** | **IDI (95% CI)** | **Test statistic** | **p Value**** |
| --- | --- | --- | --- | --- | --- | --- |
| IHD |  |  |  |  |  |  |
| NORRISK-2 | 0.75 (0.71-0.79) | N/A | N/A | N/A | N/A | N/A |
| RF | 0.87 (0.85-0.89) | Ref. | Ref. | Ref. | Ref. | Ref. |
| wTPT global | 0.86 (0.83-0.88) | N/A | N/A | N/A | N/A | N/A |
| wTPT + RF | 0.89 (0.87-0.91) | < 0.001 | 0.71 (0.57-0.84) | 0.058 (0.044-0.071) | 121.1 | < 0.001 |
| wTPT carotid | 0.79 (0.75-0.82) | N/A | N/A | N/A | N/A | N/A |
| wTPT carotid +  RF | 0.88 (0.85-0.90) | 0.021 | 0.39 (0.25-0.52) | 0.020 (0.012-0.029) | 46.9 | < 0.001 |
| wTPT femoral | 0.85 (0.82-0.88) | N/A | N/A | N/A | N/A | N/A |
| wTPT femoral +  RF | 0.89 (0.87-0.91) | < 0.001 | 0.67 (0.53-0.80) | 0.051 (0.039-0.063) | 112.3 | < 0.001 |
| IS |  |  |  |  |  |  |
| NORRISK-2 | 0.68 (0.61-0.74) | N/A | N/A | N/A | N/A | N/A |
| RF | 0.79 (0.74-0.84) | Ref. | Ref. | Ref. | Ref. | Ref. |
| wTPT global | 0.78 (0.72-0.84) | N/A | N/A | N/A | N/A | N/A |
| wTPT + RF | 0.83 (0.78-0.88) | 0.0086 | 0.58 (0.31-0.85) | 0.011 (0.0044-0.018) | 22.5 | < 0.001 |
| wTPT carotid | 0.73 (0.66-0.80) | N/A | N/A | N/A | N/A | N/A |
| wTPT carotid +  RF | 0.82 (0.77-0.86) | 0.079 | 0.41 (0.13-0.68) | 0.0062 (0.0011-0.011) | 16.2 | < 0.001 |
| wTPT femoral | 0.76 (0.70-0.82) | N/A | N/A | N/A | N/A | N/A |
| wTPT femoral +  RF | 0.82 (0.77-0.87) | 0.035 | 0.36 (0.087-0.63) | 0.0073 (0.0027-0.012) | 16.5 | < 0.001 |

*p value compared with risk factors

**p Value Likelihood ratio test

IDI = integrated discrimination improvement. IHD = ischemic heart disease. IS = ischemic stroke. NRI = net reclassification improvement. IDI = integrated discrimination improvement. RF = risk factors, including sex, age, dyslipidemia, hypertension, past or current smoking, diabetes, family history and elevated Lp(a). Test statistics = likelihood ratio test statistics. wTPT = weighted total plaque thickness.

## Supplemental Table 6. Incremental Value of wTPT to Risk Factors in Discriminating CV Disease - Stratified by Sex

|  | **Females** |  | **Males** |  |
| --- | --- | --- | --- | --- |
| **Variable** | **c-statistic (95% CI)** | **p Value*** | **c-statistic (95% CI)** | **p Value**** |
| CV Disease |  |  |  |  |
| NORRISK-2 | 0.70 (0.64-0.77) | N/A | 0.70 (0.66-0.74) | N/A |
| RF | 0.83 (0.79-0.87) | Ref. | 0.83 (0.79-0.86) | Ref. |
| wTPT | 0.81 (0.75-0.87) | N/A | 0.84 (0.81-0.86) | N/A |
| wTPT + RF | 0.89 (0.84-0.92) | 0.001 | 0.86 (0.83-0.89) | < 0.001 |
| IHD |  |  |  |  |
| NORRISK-2 | 0.72 (0.63-0.81) | N/A | 0.70 (0.66-0.74) | N/A |
| RF | 0.88 (0.84-0.93) | Ref. | 0.82 (0.79-0.86) | Ref. |
| wTPT | 0.81 (0.72-0.90) | N/A | 0.84 (0.81-0.86) | N/A |
| wTPT + RF | 0.90 (0.86-0.95) | 0.11 | 0.86 (0.83-0.88) | < 0.001 |
| IS |  |  |  |  |
| NORRISK-2 | 0.72 (0.63-0.81) | N/A | 0.70 (0.61-0.79) | N/A |
| RF | 0.83 (0.79-0.88) | Ref. | 0.79 (0.72-0.86) | Ref. |
| wTPT | 0.81 (0.72-0.90) | N/A | 0.80 (0.73-0.87) | N/A |
| wTPT + RF | 0.87 (0.82-0.93) | 0.084 | 0.83 (0.76-0.90) | 0.12 |

*p value compared with risk factors

**p Value Likelihood ratio test

CV = cardiovascular. IHD = ischemic heart disease. IS = ischemic stroke. RF = risk factors, including sex, age, dyslipidemia, hypertension, past or current smoking, diabetes, family history and elevated Lp(a). Test statistics = likelihood ratio test statistics. wTPT = weighted total plaque thickness.
